# Elementary nervous systems

**DOI:** 10.1098/rstb.2020.0347

**Published:** 2021-03-29

**Authors:** Detlev Arendt

**Affiliations:** ^1^ Developmental Biology Unit, European Molecular Biology Laboratory, Heidelberg, Germany; ^2^ Centre for Organismal Studies, University of Heidelberg, Heidelberg, Germany

**Keywords:** nervous system origins, neuron evolution, nervous system evolution

## Abstract

The evolutionary origin of the nervous system has been a matter of long-standing debate. This is due to the different perspectives taken. Earlier studies addressed nervous system origins at the cellular level. They focused on the selective advantage of the first neuron in its local context, and considered vertical sensory-motor reflex arcs the first nervous system. Later studies emphasized the value of the nervous system at the tissue level. Rather than acting locally, early neurons were seen as part of an elementary nerve net that enabled the horizontal coordination of tissue movements. Opinions have also differed on the nature of effector cells. While most authors have favoured contractile systems, others see the key output of the incipient nervous system in the coordination of motile cilia, or the secretion of antimicrobial peptides. I will discuss these divergent views and explore how they can be validated by molecular and single-cell data. From this survey, possible consensus emerges: (i) the first manifestation of the nervous system likely was a nerve net, whereas specialized local circuits evolved later; (ii) different nerve nets may have evolved for the coordination of contractile or cilia-driven movements; (iii) all evolving nerve nets facilitated new forms of animal behaviour with increasing body size.

This article is part of the theme issue ‘Basal cognition: multicellularity, neurons and the cognitive lens’.

## 1. Introduction

Reflecting on the benefits that came along with the evolution of the nervous system, a straightforward answer is that it boosted cognition—not the least in ourselves. The more complex a nervous system, the higher its cognitive capacities. When it comes to the very origins of the nervous system, however, this link is less clear. It may seem surprising at first, but major questions about nervous system origins remain unsolved, starting with the most basic: what exactly was it that from then onwards deserved to be called a nervous system? What was the major innovation? What became possible that was not possible before?

**Figure 1.**
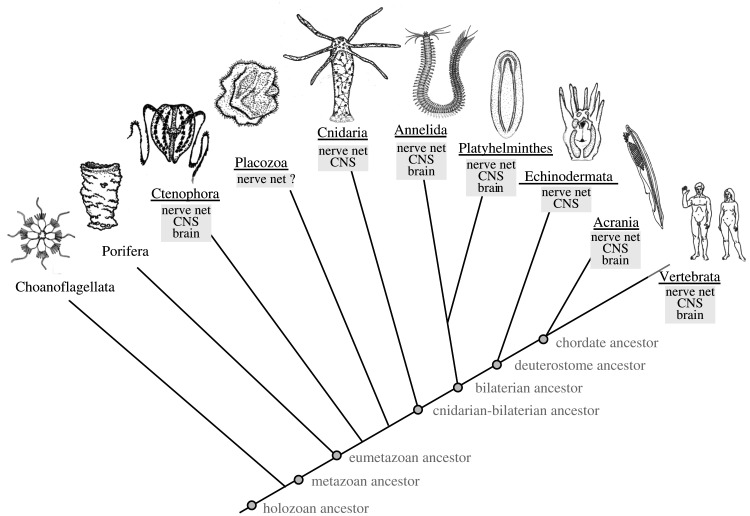
A simplified phylogenetic tree of the animals. Depicted species represent groups of special relevance for comparative neurobiology that are mentioned in the text. The presence of a centralized nervous system in cnidarians and of a brain in ctenophores is discussed in Satterlie [1] and Jager *et al*. [2]. The branching of the tree follows Kapli & Telford [3].

Crucially, the answer to these questions is *not* that the nervous system enabled animal cognition for the first time. As is clear most recently with this special issue of *Transactions*, most of the basic elements of cognition were already present and functional before the nervous system evolved. The ability to selectively perceive specific stimuli, the discrimination between favourable and unfavourable, the assessment of the overall valence of a situation, the retention of memory, and the integration of information for decision-making—all of this was in place in one form or another in unicellular organisms and early metazoans that did not (yet) possess a nervous system. In consequence, what *did* the nervous system enable? A first answer can be easily framed: nervous system evolution is about information exchange and integration *between* cells. It is about shifting cognition from the unicellular to the multicellular level; it is about the evolution of circuits. But what was the nature of the first circuit, the elementary circuit, and what did it achieve? What new functionality was added to the animals' toolbox that made them thrive in the Precambrian past? Working on diverse bilaterian and non-bilaterian metazoan animals without or with simple nervous systems (figure 1), comparative neurobiologists have addressed these questions for the past 150 years and provided manifold answers. I will survey their contributions and the vivid debate on nervous system origins and, building and expanding on this, attempt some preliminary conclusions.

The divergent historical viewpoints are best understood if one considers that they were looking at nervous systems at different levels—at the cellular level at first, and then at the level of entire tissues or even the whole body. The cellular perspective—i.e. the origin of the first neuron—was developed as early as the late nineteenth century by Kleinenberg [4] and the Hertwigs [5], and later refined by Parker [6]. These authors derived the first neurons from isolated cells that started to relay to each other and thus formed *elementary circuits*, mediating vertical information flow from receptor to effector cells. Such circuits would enable improved integration and processing of environmental signals and thus enhance and diversify basic forms of cognition in early animals. The tissue perspective—the origin of the nervous system as a whole—was developed in the middle of the twentieth century by Pantin [7] and Passano [8], and further elaborated by Mackie [9] and Pavan de Cecatty [10]. Rather than on isolated vertical circuits, these authors focused on the evolutionary emergence of *elementary nerve nets* that interconnected receptor cells and/or effector cells horizontally across entire tissues; and addressed the advantages this brought to the functioning of the animal body as a whole. Such early nerve nets would have facilitated coordination and integration of primordial behaviours.

Beyond that, the hypotheses on nervous system origins differ in the nature of the effectors that were envisaged downstream of the elementary circuits or nerve nets. While most of the twentieth-century authors favoured contractile effector cells or tissues, more recent contributions also considered bands or sheets of ciliated cells as primordial effectors, for the transport of food or locomotion [11]. Others envisaged effector cells carrying out immune functions in response to environmental microbes [12]. Finally, a strong camp emphasized the secretory nature of early neurons that may have acted at a distance on effector cells via the release of neuropeptides [13–15].

For each view, the underlying assumptions on the relatedness of neurons to other cell types will be discussed and evaluated from a modern viewpoint—taking into account cross-phyla comparisons of neural cell types and tissues [16–29], as well as of their constituent molecular machinery such as synaptic proteins, ion channels and transmitter systems [30–35].

From this survey, some consensus emerges. As advocated by the twentieth-century comparative neurobiologists, the new functionality that came with the nervous system may indeed have been most apparent at the tissue level—with a nerve net as a whole-body integrative system. Nascent nerve nets may have coordinated body movements—involving contractions of tissue sheets for rapid shape changes, or ciliary beating across tissues for feeding and locomotion. Either option finds support in recent single-cell transcriptomics-based, whole-body cell type and tissue comparisons; and both inventions would have been especially relevant in animals of increasing body size. This suggests that the non-neural-to-neural transition may have occurred more than once, in different tissues and, possibly, distinct evolutionary lineages.

## 2. Elementary circuits: simple sensory-effector reflex arcs

The early cell-centric views on nervous system evolution focused on the emergence of the first neuron as the key element of a local vertical circuit, which relays information between sensory receptor and effector cells. From this perspective, major questions can be put as follows: what was the sensory receptor and what the effector cell that formed part of the first elementary circuit? Epithelial, and different sensory and contractile cell types have been put forward in this context since the nineteenth century, and new candidates have been added in more recent times—such as ciliated cells, secretory cells or even immune cells.

### (a) Kleinenberg's neuromuscular theory

**Figure 2.**
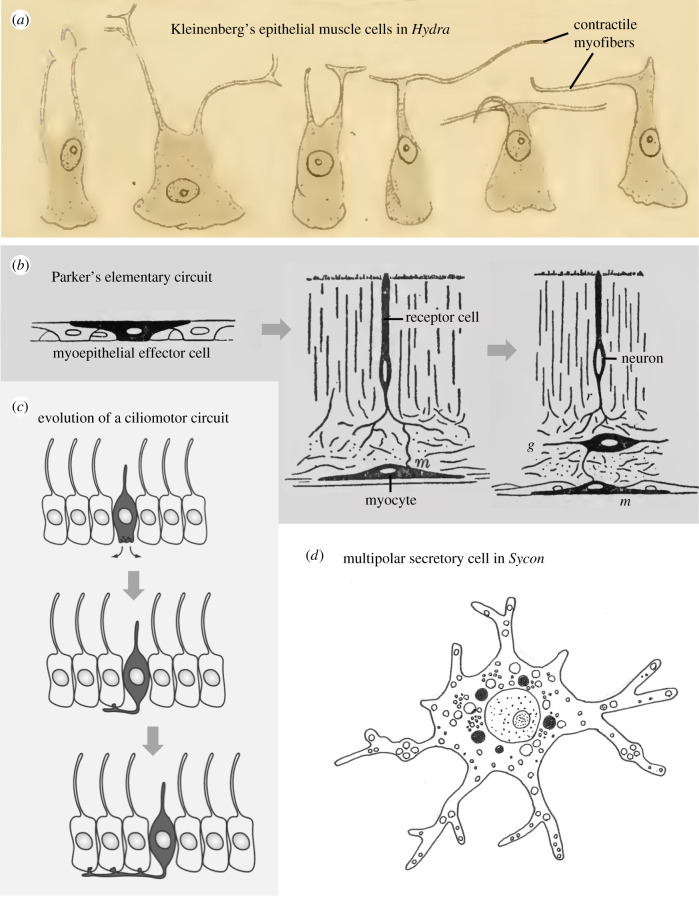
Historic views of elementary sensory-effector circuits. (*a*) Kleinenberg's observation of epithelial muscle cells in *Hydra*. The first circuit would have evolved via the physical separation of the contractile myofiber from the cell body. Original drawings from Kleinenberg [4]. (*b*) Parker's three stages of elementary circuit evolution. Original drawings from Parker [6]. (*c*) Three steps towards the evolution of a simple ciliomotor circuit according to Jékely [11]. (*d*) A multipolar secretory cell filled with dense core vesicles and multiple extensions as observed in nervous system-less sponges. Redrawn after Lentz [36]. (Online version in colour.)

Nikolaus Kleinenberg was the first to come up with ideas on how neurons and primordial circuits emerged in evolution [4]. Studying the epithelial muscle cells in the cnidarian fresh water polyp *Hydra*, he observed that these cells possessed contractile myofibers that were connected to the rest of the cell via slender processes (figure 2*a*)—as if the cell was subdivided into two separate functional compartments: a contractile fibre and the cell body proper (which Kleinenberg considered sensory given its prominent cilium). In his *neuromuscular theory*, Kleinenberg thus assumed that the neuron and the muscle cell of the first elementary circuit originated from an evolutionary precursor cell that was both excitable and contractile, resembling *Hydra*'s epithelial muscle cells. This ancient multifunctional cell would have physically segregated the excitable from the contractile part, so that both formed separate entities. He thus put forward an early (and with today's knowledge impossible) version of a division of labour scenario of cell type evolution [18,37]. Kleinenberg's early version of the neuromuscular theory was refuted by his contemporaries [5]. The brothers Richard and Oscar Hertwig considered *Hydra*'s myoepithelial cells to be simple contractile cells with an epithelial anchor, which coexisted with separate sensory and ganglion cells in the same epithelium. They thus believed that these cell types evolved independently, each one on their own and from epithelial cells, and not from multifunctional precursors.

### (b) Parker's independent effectors

In his influential monograph on the ‘elementary nervous system’, George Howard Parker followed the viewpoint of the Hertwig brothers [6]. Parker postulated three steps towards the evolution of the first neuron (figure 2*b*). At first, some kind of independent effector cells were scattered across ancient epithelia, possibly resembling Kleinenberg's myoepithelial cells or nematocysts in cnidarians. These effector cells were supposed to react to stimuli autonomously. Secondly, separate receptor cells were assumed to have evolved from the undifferentiated epithelium adjacent to the effector cells. ‘The most primitive nerve cell from the standpoint of animal phylogeny is the sense-cell, or receptive cell, such as occurs in the sensory epithelium of the coelenterates’ [3, p. 210]. As a third step, real neurons evolved between receptor and effector to eventually give rise to the reflex triad of ‘receptor, adjustor and effector’. Similar ideas were voiced by Cajal [38] who proposed an ideal invertebrate in which independent neurons are scattered across epithelia and are—each one of them—both sensory and motor. Thus, the founders of modern neurobiology proposed that the most elementary form of the nervous system was a characteristic mononeuronal reflex arc composed of a sensory neuron, a neuron and an effector cell [10]. In line with these ideas, two- or three-celled mechanosensory-contractile vertical neuronal circuits are widespread in today's cnidarians [39] and ctenophores [40].

### (c) Elementary ciliomotor circuits

A modern variant of Parker's theory put forward by Gáspár Jékely differs in the nature of the effector cells, interpreted as epithelial cells with motile cilia [11]. This view links the evolutionary emergence of neurons to the emergence of a primordial ciliomotor circuit for the improved coordination of ciliary swimming. Following this view, the evolution of neurons started from a sensory cell that slowly acquired basal processes, which contacted neighbouring cells bearing motile cilia (figure 2*c*). Indeed, ciliary bands with motile cilia coordinated by sensory-ciliary mini-circuits are a widespread means of locomotion in primary larvae, innervated by an apical organ and associated receptor cells that mediate mechano-, chemo, baro- or photosensory input [41–44]. Of note, such ciliomotor larval nervous systems are only reported for bilaterians.

Similarly, sensory-ciliomotor circuits drive ciliary swimming in the enigmatic ctenophores (figure 1). In these animals, an apical sense organ innervates and controls the rhythmic beating of the comb plates, which are composed of motile cilia [45,46]. Importantly, however, the sensory-ciliomotor circuits of bilaterians and ctenophores are often regarded independent evolutionary acquisitions ([46]; see however [47]).

### (d) The first neuron—a secretory cell?

Parker and followers emphasized the sensory nature of the first neuron, and regarded it the sister cell type of sensory epithelial cells. Implicit to this view, these sensory cells would have started secondarily to emit signals to the neighbouring effector cells via secretion. Other authors turned this view around and instead assumed that the secretory nature of the neuronal precursors was first and the sensory nature secondary. They thus considered the sister cell type of the first evolving neuron a secretory cell [48–50]. Consequentially, neurons would have first appeared as neurosecretory cells. For example, studying the subcellular localization of catecholamines, serotonin, neuropeptides and other putative transmitters in sponges, Thomas L. Lentz observed bi- and multipolar secretory cells (figure 2*d*) that he likened to primitive neurons [36]. In nervous system evolution, similar excitable and conductive ‘prenervous cells’ with secretory capabilities would have influenced nearby effectors; they would have developed elongated processes, become sensitive to stimuli and thus given rise to the first circuits [50]. Studying neurosecretion in *Hydra*, Lentz pointed out the essential value of neurosecretion not only for the chemical synapses but also for the activity of nervous tissue as a trophic system, that is, controlling growth and development by means of synthesis and release of specific substances [10,50].

### (e) An ancestral neuroimmune system

Related to the notion of early neurons serving secretory functions is the more recent idea that neurons may have evolved as immune cells [12]. Given that multicellular animals emerged in a world of microbes, and that all extant animals are colonized by a large number of symbiotic microbes; and considering that host-associated microbiota has been shown to be in a permanent dialogue with the host enteric and central nervous systems [51], neurons might have emerged as a cell type exerting functions commonly attributed to immune systems: they may have monitored the environment and sensed and discriminated microbes. Indirectly, via secretory release, and directly, via innervation, neurons may have adjusted the animal's vital processes (i.e. development, physiology, tissue homeostasis and behaviour) to the presence and state of the microbiota. In addition, neurons might have exerted an immunomodulatory effect by tuning the immune response of epithelial cells [12]. Later in evolution, the intercellular communication channels established by such neuroimmune cells could then have acquired secondary functions in the form of sensory-motor circuits.

### (f) Local circuits first or nerve net first?

In summary, the above cell-centric views envisage the first neuron as the key part of an elementary sensory-effector circuit. They start from more or less isolated precursors, as is the case for Kleinenberg's myoepithelial cells, Parker's effectors, Lentz' neurosecretory cells and for the putative neuroimmune cells. Each of these can be seen as stand-alone sensory-effector reflex arcs that enhance local integration by means of their newly acquired neurite-like processes. Only in a second step would these elementary circuits get horizontally interconnected.

What would have been the selective advantage of such local circuits? This question has been extensively discussed and is far from trivial [7,9,11,52]. One elegant concept envisages an increase in ‘sensory-to-motor transformation’, defined as the ratio of involved sensory cells to effector cells that they can influence [11]. Another advantage would lie in the improved conductive capacity of the newly evolving neurites, which may have enabled faster information processing and integration. Such changes would entail incremental increases in cognitive power for the animal.

Alternatively, isolated vertical elementary circuits may have never existed. Instead, early neurons may have been horizontally interconnected from the very beginning, across tissues, in the form of a nerve net. In these nets, vertical and horizontal information transfer may have co-occurred, with dispersed receptor cells feeding into the nerve net and distributed effector tissue innervated by the nerve net. Specialized local circuits would then have arisen via restricted secondary diversification. This exciting alternative requires us to change perspective: from the cell to the tissue level. This way, the selective advantage of the nascent nervous system becomes more obvious.

## 3. Elementary nerve nets (i): the contractile network hypothesis

**Figure 3.**
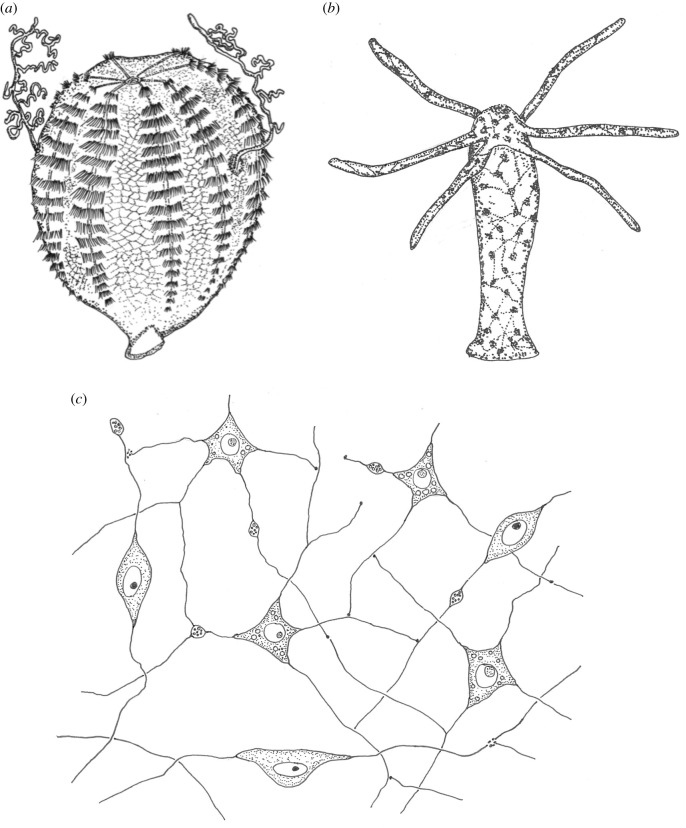
Characteristic nerve nets in ctenophores and cnidarians. (*a*) The polygonal epithelial nerve net of *Pleurobrachia pileus* redrawn after Jager *et al*. [45]. (*b*) The epithelial nerve net of the *Hydra* polyp from Arendt [53]. (*c*) Cellular view of the *Hydra* nerve net. Nerve nets are known to contain stereotypic elements with distinct transmitters [54]. Redrawn after Lentz [50].

In search of the first evolutionary manifestation of the nervous system, some authors in the mid twentieth century no longer envisaged local, vertical circuits. Instead, they postulated the primacy of the *elementary nerve net*: i.e. neurons forming large horizontal networks spanning entire tissues from the very beginning. Nerve nets as propounded by these views are observed for example in extant ctenophores and cnidarians (figure 3). A primordial nature of the nerve net would require that some kind of tissue- or body-wide system predated the nervous system, which then evolved into the nerve net. If so, what was the nature of this system and what was its function? Or, in other words: what kind of cellular network was the evolutionary precursor of the nerve net, and can we identify related non-neural networks (‘sister networks’) in extant animals?

### (a) Muscle fields and global shape changes

An early tissue-centric view was developed by Carl Frederick Abel Pantin [7], who was the first to postulate an elementary nerve net (and considered local neural circuits secondary specializations thereof). Pantin suggested that the nerve net evolved alongside an epithelial contractile tissue sheet, conducting excitation with its longer processes faster than the contractile sheet cells themselves. This led to simultaneous contraction of the entire tissue as opposed to the slower wave-like contractions. The coordinated contraction of contractile tissue units (referred to as muscle fields; [10]) enabled global shape changes and behaviour that was not possible before. This was especially relevant for animals with increasing body size, which needed to respond to environmental stimuli with a total (rather than local) integration of effectors—which cannot be achieved by isolated reflex arcs [7].

Pantin built his theory on observations of contractile systems in cnidarians, referring for example to the sphincter closure apparatus in *Calliactis*. He reasoned that, in many cases, the activity of the nerve net would result from spontaneous, endogenous activity, as is also frequently observed in other cnidarians (see for example [55]). The response to an environmental stimulus would then consist of a prolonged change in the pattern of spontaneous activity (rather than the initiation of activity itself). Such behaviour would be generated internally and modified by external cues—with the spontaneous pattern having priority over that of the reflex pattern: ‘The reflex arc is not a primitive unit’ [4, p. 176].

**Figure 4.**
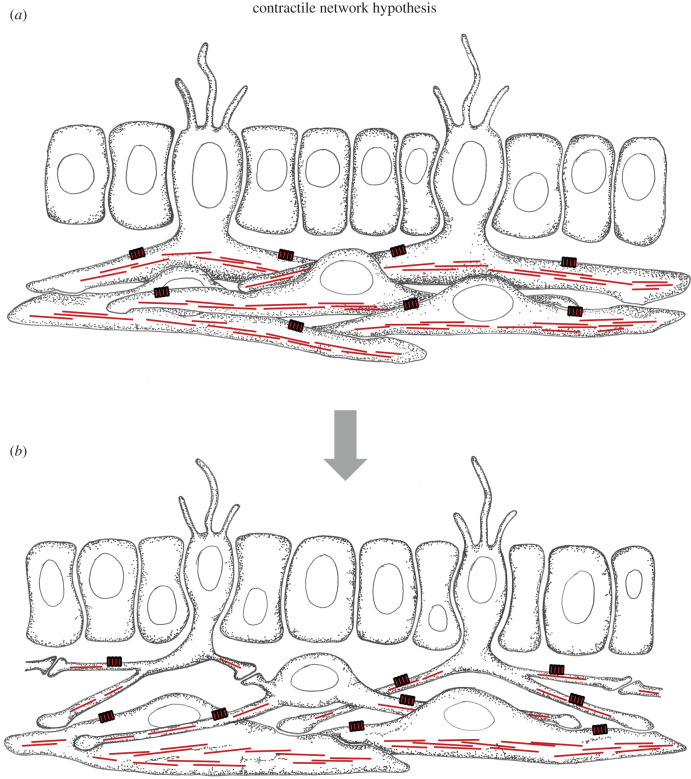
The contractile network hypothesis. (*a*) Evolutionary precursor state with epithelial mechanosensory and mesenchymal cells with long interconnected contractile and conductive processes forming a tissue-wide network. (*b*) The first nervous system comprising mechanosensory neurons innervating a tissue-spanning elementary nerve net composed of multipolar interneurons. The nerve net neurons innervate a network of contractile myocytes. Red boxes on the cells represent conductive ion channels. Red lines indicate actomyosin filaments for contraction. (Online version in colour.)

Inspired by Pantin's theory, the cnidarian biologist L. M. Passano developed a tissue-centric division of labour scenario with nerve nets and muscle sheets diversifying from a single network of highly interconnected, fibrous cells with contractile and conductive properties [8]. He proposed that some of these cells acquired the capacity to endogenously generate electric activity, comparable to pacemaker cells, while others started to respond to these protoneurons. The former then specialized more and more on the generation, integration and conduction of electrical signals and finally became the neurons of the first nerve net, whereas the letter specialized on contraction and became bona fide muscle cells innervated by the nerve net. Passano's idea is named here the ‘contractile network hypothesis’, which can be regarded a modern tissue variant of the initial neuromuscular theory. It is visualized in an interpretative drawing in figure 4.

Another cnidarian biologist, George O. Mackie, strengthened the case for this hypothesis by describing various forms of contractile and/or conductive tissue sheets in cnidarians [9]. For example, the bell-shaped body of the hydromedusae is composed of myoepithelial cells, which constrict to produce the locomotory jet of water. The excitation for this response is conducted in the contractile sheet itself. In addition, the contractile sheet is innervated by the ganglion cells of a proper nerve net, which act as pacemakers initiating the rhythmical swimming beat and rapidly transmitting the excitation into all four quadrants of the medusae [9]. In general, multifunctional contractile and conductive tissue sheets appear typically involved in simple behaviours such as rapid whole-tissue contraction, whereas functionally separate nerve net and muscles are involved in more complex movements that require sophisticated integration. Thus, neuroid-myoid tissues (resembling the presumed multifunctional precursor tissue), as well as bona fide nerve nets and muscle sheets (representing the possible outcome of an evolutionary division of labour process) coexist in cnidarians. This makes the contractile network hypothesis a plausible scenario that may occur whenever complex behaviour evolves in animals of increasing body size.

The contractile network hypothesis also underlies the so-called skin brain thesis recently put forward by Fred Keijzer and colleagues [56,57]. In line with Pantin and Passano, they postulate that early nervous system evolution gave rise to a nerve-net-innervated muscle effector tissue, the primary source of animal motility. This tissue was capable of inducing and coordinating self-organized contractile activity across an extensive muscle surface underneath the skin [56,58].

### (b) A neuromuscular orthogon in *Dickinsonia*?

**Figure 5.**
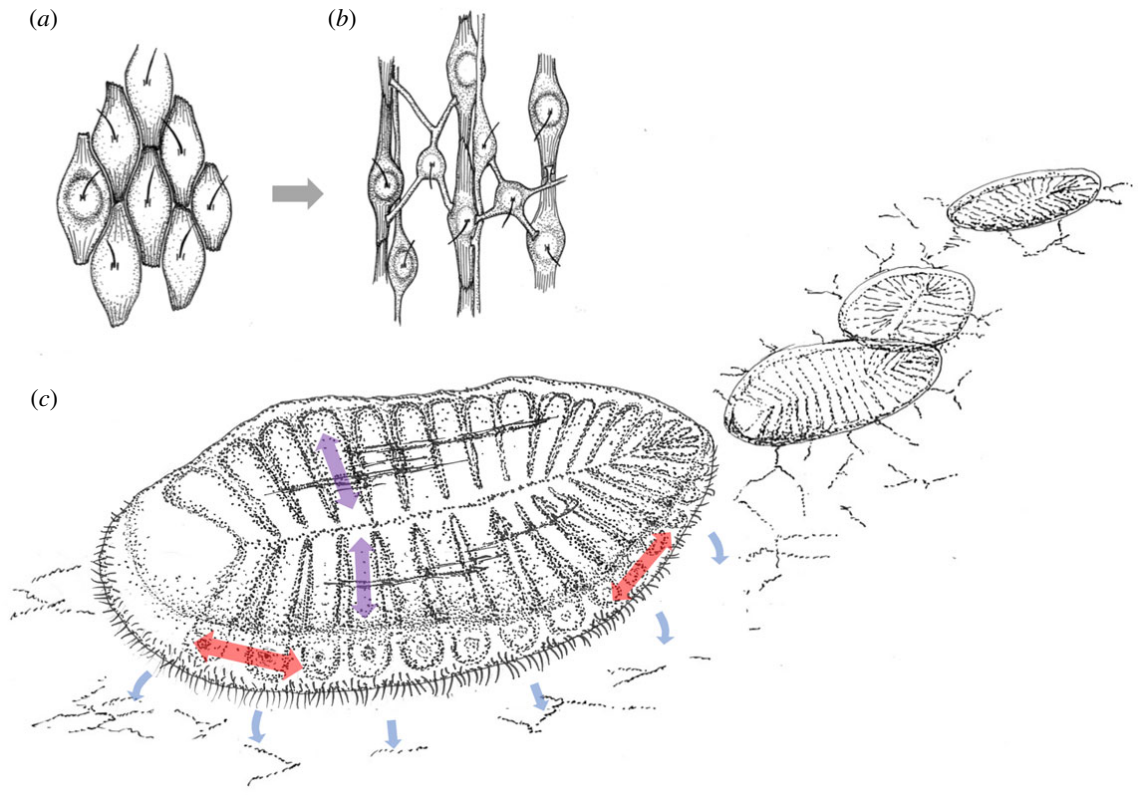
Locomotor patterns in ancestral metazoans. (*a*,*b*) The evolution of nerve-net innervated longitudinal musculature from polarized conductive-contractile cells via division of labour. From Arendt *et al.* [37]. (*c*) Interpretative drawing of a *Dickinsonia*-like animal feeding on organic mats covering the Ediacaran seafloor ('old elephant skin'), following Evans *et al.* [62] and Ivantsov [63]. Fossil evidence indicates that the animals remained stationary for a period of time, removed the organic mat beneath them via external digestion or ciliary activity, and then moved from that area leaving a depression ('footprint'). Chains of footprints are interpreted as forward movement. Wrinkles on the surface indicate the presence of longitudinal muscles parallel or perpendicular to the gastric pouches (violet and red double arrows), enabling shape change. Locomotor movements may have been cilia- and musculature-driven and controlled by nerve nets.

In support of the contractile network hypothesis, the presence of a well-developed nerve net in cnidarians and in ctenophores reliably correlates with the presence of myofibers directly innervated by the nerve net neurons [37,52,59]. In these animals, muscular systems are composed of longitudinal muscles (in the direction of the primary body axis) and of ring muscles [60,61]. This indicates that, once myofibers segregated from neurons, they were arranged at right angles and contracted antagonistically, in an arrangement termed a *neuromuscular orthogon* (figure 5*a*,*b*) [37]. In line with this, forward locomotion of the Ediacaran fossil *Dickinsonia* has recently been discussed based on body and trace fossils, and may have involved antagonistic contraction of myofibers oriented parallel and perpendicular to the longitudinal axis [62]. Indicative of this, the upper surface of these fossils frequently contains wrinkle marks parallel to the longitudinal axis (figure 5*c*). These observations constitute a plausible anatomical setting in which the nervous and muscular system may have co-evolved.

### (c) Contractile-conductive tissue in sponges

**Figure 6.**
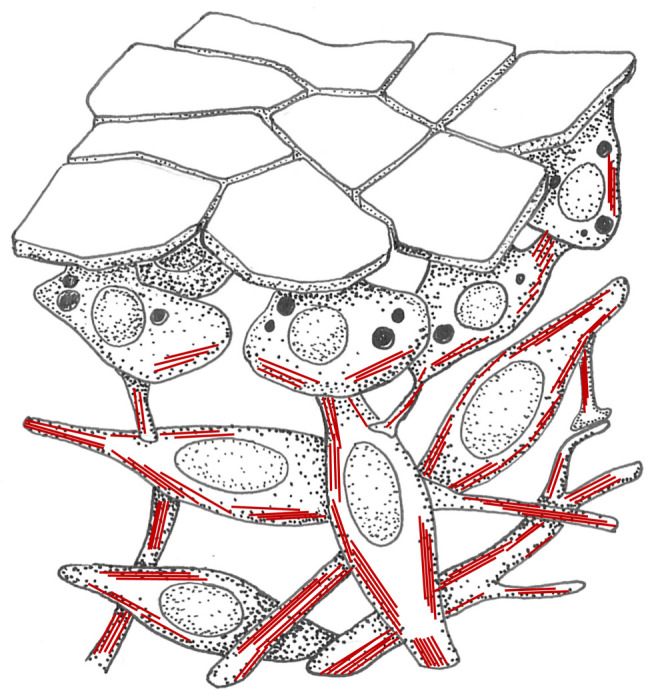
Ectomesenchyme in sponges. Interconnected contractile and conductive cells with secretory granules form a mesenchymal network underneath the pinacocyte outer epithelium. Red lines indicate actomyosin fibres. Redrawn and modified after Pavans de Ceccatty [10].

How about early-branching Metazoa that do not (yet) have a nervous system, such as sponges—do we find interconnected myofiber-like cells as postulated by the contractile network hypothesis? The sponge biologist Max Pavans de Cecatty affirmed this, investigating various sponges [10]. He showed that the sponge ectomesenchyme represents neuroid-myoid tissue with mixed contractile and conductive properties. Its surface comprises flat expansions of so-called pinacocytes, the cell bodies of which are located deeper in the connective tissue, where contractile cells form a mesenchymal network—connected to each other and to the pinacocytes (figure 6). All cells have secretory granules, supposedly for cell–cell communication. Based on these observations, Pavans de Cecatty regarded the sponge contractile mesenchyme a ‘protonervous or neuroid system’. ‘Reticulated and discrete, it has pacemaker and secretory activities, is directly excitable, and is conductive from cell to cell’ [7, p. 386].

## 4. Elementary nerve nets (ii): the neurosecretory network hypothesis

The contractile network hypothesis builds on the premise that first coordinated animal body movement was driven by tissue contraction. Yet, this is not the full picture. In an alternative view, larger fields of motile cilia might have propelled early animals forwards [63]—and myofibers may have mediated steering rather than propulsion. Indeed, the Ediacaran *Dickinsonia* are postulated to have possessed a ventral mucociliary sole for particle transport, ciliary gliding or even swimming movements [37,63,64]. *Dickinsonia* and related species such as *Yorgia* apparently moved forward by short episodes of swimming as evidenced by a series of feeding traces on algal mats without any evidence of the body moving on the substrate (figure 5*d*,*e*). Just like tissue contraction, ciliary beating patterns that may have enabled such swimming movements would have required increasing degrees of coordination with increasing body size.

A concurrent scenario for nerve net evolution at the tissue level thus gains momentum: namely that ciliated tissue with coordinated beating was centre stage in nerve net evolution. A homogeneous array of neuroid-ciliated cells may have been in place in early metazoans—before a division of labour event separated neuronal precursors and motile ciliated sister cell types. How can we envisage a possible evolutionary emergence of a nerve net from within ciliated tissue?

### (a) The neurosecretory network hypothesis

**Figure 7.**
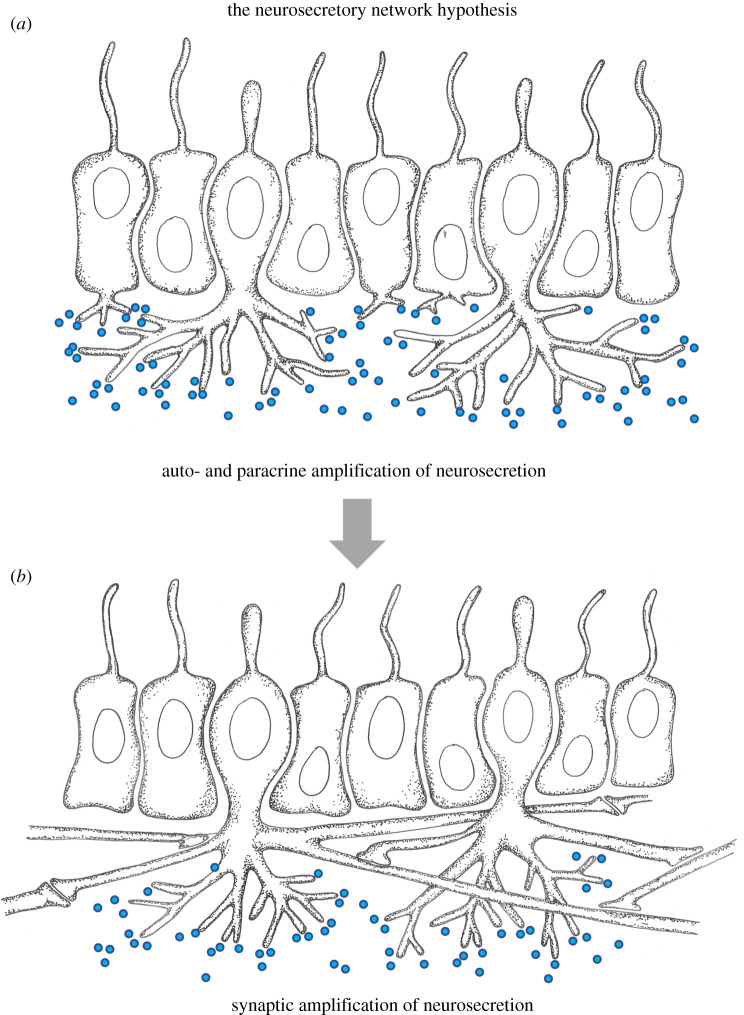
The neurosecretory network hypothesis. (*a*) Evolutionary precursor state. Ciliated tissue with equally spaced sensory-neurosecretory cells that secrete neuropeptides. Sensory-neurosecretory cells form numerous basal projections. (*b*) Elementary nerve net. Horizontal projections of sensory-neurosecretory interconnected via synapses. Synaptic amplification of neurosecretion. Blue circles indicate vesicles of secreted neuropeptides. (Online version in colour.)

This hypothesis builds on the primacy of secretory cells as advocated by Lentz and others (see above), and is put forward for the first time in an elaborate fashion by Gáspár Jékely in this issue of *Transactions* [15]. Here, nervous system evolution starts from a sheet of ciliated cells. Initially, cilia are both sensory and motile and respond to environmental cues autonomically with changes in their beating pattern. Enhanced synchronization between cells is then achieved via the basal release of neuropeptides that trigger autocrine and paracrine amplification. Via division of labour, some of these cells specialize in sensory perception and neuropeptide release and become sensory-secretory cells interspersed among ciliary effector cells, as depicted in figure 7*a*. In this arrangement, all tissue cells would be linked up into a chemical network made up of diffusible neuropeptides [15]. Yet, signalling via diffusion of peptides becomes inefficient in larger bodies. This prompts the gradual horizontal elongation of basal secretory processes until they overlap between distant neurosecretory cells. Finally, synapses would evolve between these processes, thus interconnecting sensory-neurosecretory cells of the same type into coherent nerve nets as depicted in figure 7*b*. This way, the now physically interconnected network cells would be able to display rapid synchronized activity with pulsatile peptide release for the tissue-wide control of ciliary beating or contractions.

The neurosecretory network hypothesis finds support by the omnipresence of complex peptidergic signalling in all animals except sponges [65–67], and by the dispersed and widespread occurrence of sensory-neurosecretory cells in cnidarian nerve nets [49,50] (see above) and in the echinoderm central nervous system [68]. While it is conceivable that such neurosecretory nerve nets initially controlled ciliary beating patterns, they might have started to concomitantly control the behaviour of adjacent contractile tissues.

In line with this hypothesis, Béla Vigh and Ingeborg Vigh-Teichmann showed that the chordate neural tube harbours so-called central spinal fluid (CSF)-contacting neurons. These are sensory-neurosecretory cells with ciliated apical sensory protrusions that project into the spinal fluid [13,14] and with basal secretory processes that terminate on the basal lamina. Similar cells are observed along the entire spinal cord in the basal chordate amphioxus [13]. Given that all epithelial cells of the chordate neural tube bear motile cilia, such distributed and interconnected neurosecretory cells may have initially controlled ciliary beating within an ancestral mucociliary sole [37]. Later on, these cells would have started influencing contraction of the adjacent longitudinal musculature—first in a paracrine manner, and finally via the more targeted synapses. In support of this scenario, the neuromuscular junctions of the amphioxus ventral motor roots have evolved across the neuroectodermal basement membrane [69]. Hence, a neurosecretory nerve net controlling ciliary beating of a mucociliary epithelium may have represented the starting material for the evolution of the chordate neural tube [37].

### (b) Neurosecretory centres: the apical nervous system

Besides expanding into a neurosecretory network, there is a second strategy for sensory-neurosecretory cells to maintain and enhance signalling efficiency, and to reduce the constraint imposed on chemical signalling by diffusion in an ever-increasing animal body. Concomitant with the advent of circulatory systems, these cells can start secreting neuropeptides, henceforth called hormones, into the body fluid. To this end, their secretory endings gather into a prominent plexus, which is referred to as the neurosecretory centre or neurohemal organ [15].

**Figure 8.**
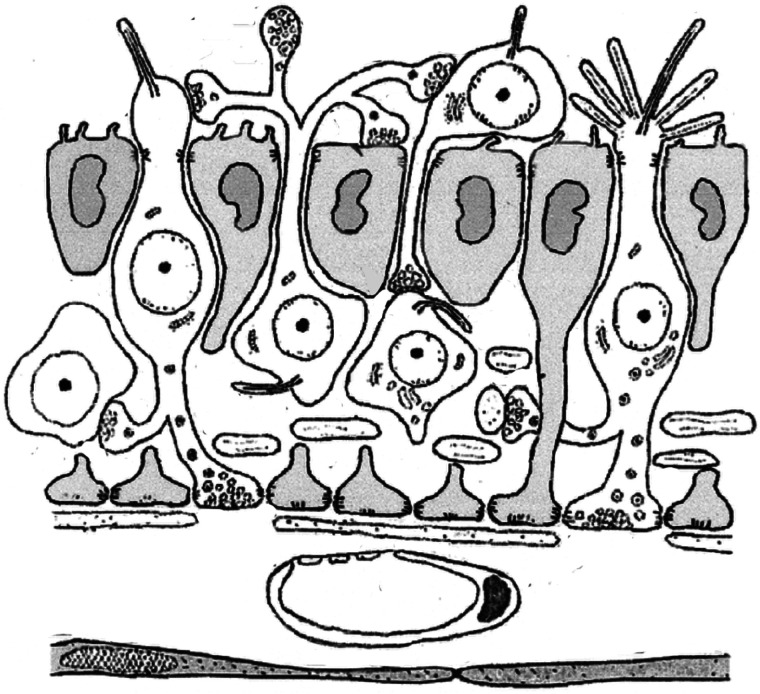
Sensory-neurosecretory cells in the vertebrate brain. Protoneuron-like cells from part of the periventricular ependyme with sensory endings responding to light, ions and flow. Basal neurosecretory processes release vesicles into external body fluids. Reproduced from Vigh *et al.* [14].

Sensory-neurosecretory cells are found in the nervous system of annelids [70] and other protostomes [71], and in the vertebrate hypothalamus (figure 8). In view of their possible ancestral nature, these cells are deemed protoneurons [13,14]. They bear various secretory and synaptic endings, which may reflect their evolutionary transition state between non-neural and neural cells. We have postulated that the so-called *apical nervous system* represents an ancient neurosecretory centre that became part of the evolving bilaterian brain [47]. This brain centre is still predominantly chemically wired and coexists with the synaptic brain in extant bilaterians [72].

All in all, with the different variants of the neurosecretory versus contractile network hypotheses we are left with concurrent and seemingly conflicting views on nervous system origins—each of them well reasoned and plausible. This prompts the question: how can we proceed from here?

## 5. Testing hypotheses via the comparison of cell type-specific molecular machinery and regulatory programmes

All of the above views on nervous system origins assume specific sister cell type relationships between neurons and other body cells. The contractile network hypothesis considers some kind of myocytes to be most closely related to neurons. In contrast, the neurosecretory network hypothesis would see secretory cells in this position. Alternatively, immune cells might be the most closely related to neurons. These hypotheses can nowadays be tested on molecular grounds.

Neurons comprise sophisticated molecular machinery, which, piece by piece, have been dissected functionally and structurally in the past decades by molecular biology and biochemistry [73,74]. For example, different kinds of chemical synapses are distinguished, such as the glutamatergic, GABAergic or cholinergic synapses. Pre- and postsynapse have been shown to be composed of multiprotein signalling complexes, and the generation and conduction of action potentials has been shown to rely on synergizing ion channels with different ion specificities [35]. This wealth of molecular knowledge on neurons and related cell types can now be harvested to test the above hypotheses on nervous system origins. The rationale is that cell types that are evolutionarily related should employ related molecular machinery, the expression of which should be controlled by similar regulatory programmes [75].

Towards this aim, the current epochal advances in single-cell genomics now allow the body-wide characterization of cell type-specific regulatory and effector genes, and thus facilitate the comparison of all cell types within and across species, and ultimately across phyla.

### (a) Striking heterogeneity of neurons

Several laboratories have pioneered whole-body single-cell sequencing for the comparative analysis of cell type inventories across metazoans [22–29]. These studies have identified neural cell type inventories in nerve-net-equipped cnidarians, ctenophores and bilaterians, and in early-branching lineages that do not possess a nervous system [25–27]. With this new comparative field just forming, first insights are apparent.

One important observation shared by all studies is the heterogeneity of neuronal cell clusters. In the ctenophore *Mnemiopsis leidyi*, synaptic scaffold components are expressed across multiple cell types, and none of them show significant co-expression of voltage-gated ion channels. This would suggest that ctenophore neurons are both diverse and of unclear relationship to those of other animals [25]. In the cnidarian sea anemone *Nematostella*, neuronal cell populations are grossly subdivided into two subsets (N1 + N2), specified by different transcription factors and representing distinct nerve nets in the tentacle ectoderm and in the body column inner layer, the gastroderm [26]. Neuronal cell types representing three distinct nerve nets likewise show divergent transcription factor identities in the cnidarian *Hydra vulgaris* [28,53]. Among bilaterians, whole-body single-cell datasets have been reported for the annelid *Platyynereis* [22] and the planarian *Schmidtea* [23,24] and likewise exhibit heterogeneous populations of neurons. As of now, it has been difficult to relate neuronal cell populations between species [25,26]—which may improve with ongoing methodological progress in the comparison of single-cell genomics datasets across larger taxonomic distances [76]. In any case, the diversity of neuronal types that is manifest in early branching metazoans would suggest that the evolutionary transition from non-neural to neural may have taken place more than once in distinct tissues—and, possibly, in distinct evolutionary lineages.

### (b) Support for the contractile network hypothesis

Given the remarkable heterogeneity of neuronal cell types—what do the single-cell datasets reveal about the relatedness of these neurons to other, non-neuronal types? Can we identify the neuronal sister cell types? And, what is more, can we identify neuron types that are more closely related to non-neuronal types than they are to other neurons? Such cases would be especially relevant to test the hypothesis of independent neuronal origins—and seem to indeed exist. For example, a recent single-cell study focusing on musculature in the sea anemone *Nematostella* reports extensive similarities between the ectodermal N1 neurons and the ectodermal myocytes of the tentacle retractor muscles—both morphologically and molecularly [29]. Unlike the mesodermal myoepithelial cells, the ectodermal myocytes detach from the epithelium into a basiepithelial position similar to the N1 ganglion neurons [77]. They form synapse-like neuromuscular junctions with postsynaptic densities and the conserved neuronal scaffolding protein Homer; and they are the only muscles to express ionotropic glutamate receptors and the neuronal RNA-binding protein *ELAV* [29]*.* Moreover, the ectodermal myocytes express the neuronal transcription factors FoxL2, SoxB2 and Sox3 [29], which they share with the N1 neurons that innervate them [26]. These data are consistent with an evolutionary kinship of the tentacle N1 neurons and retractor muscles in *Nematostella*, possibly reflecting a division of labour event as postulated by the contractile network hypothesis. Intriguingly, similarities are also apparent for the *Nematostella* gastrodermal myoepithelial cells and N2 neurons, which share combinatorial transcription factor expression involving the T-box factors *tbx1/10*, *tbx20* and the bHLH factor *hand*. In each case, the N1 and the N2 neurons appear to be more closely related to the different contractile cell types than they are to each other. To strengthen the case, it will be important to work out whether the cnidarian ectodermal and gastrodermal neuron and muscle types are conserved in the bilaterians or in the ctenophores, and whether similar cell type interrelationships hold true for these groups. Of note, within the bilaterian superphylum ectodermal muscles are only reported for a few Spiralian groups—including some annelids, molluscs and flatworms [78]. Unravelling their molecular identity and possible relatedness to cnidarian ectodermal musculature appears especially rewarding.

A kinship of glutamatergic neurons and myocytes finds support by the co-usage of postsynaptic modules such as the Homer-containing calcium-induced calcium release module and of the same conductive molecular machinery including all four Shaker potassium channel paralogs K_v_1 to 4 [35]. This suggests that these modules were already present in the contractile-conductive precursor cells as depicted in figure 4.

### (c) Support for the neurosecretory network hypothesis

Other observations in turn strengthen the view that at least subsets of vertebrate neurons may have evolved from sensory-neurosecretory cells, as discussed above. First and foremost are the many similarities that motor neurons in the ventral neural tube share with pancreatic secretory cells, including neuropeptide and neurotransmitter release, synaptic machinery, and action potentials [35,79]. Furthermore, the combination of transcription factors specifying these neurons, including the homeodomain factors *mnx*, *nk6*, *pax6* and *Islet*, and the onecut transcription factor *hnf6* [80] closely matches that of the secretory pancreatic islet cells [81]. These data indicate that both the neurons of the vertebrate ventral neural tube and foregut-derived pancreatic islet cells may be evolutionary derivatives of sensory-neurosecretory cells in a digestive mucociliary sole [37]. In line with this, a similar pancreatic/ventral neural tube-like transcription factor signature is also shared by selected groups of neurons and gut cells in the sea urchin [79], and is likewise characteristic for the pharyngeal ectoderm in the cnidarian *Nematostella* [26], which gives rise to secretory cells (and a small number of neurons alike). Furthermore, in the demosponge *Spongilla* the *nkx6+* secretory digestive choanocytes have been shown to specifically express orthologs of postsynaptic genes such as *Homer* and *Shank*, which may indicate some affinity of these cells to protoneurons [82].

## 6. Conclusion

One and a half centuries of comparative work and educated conjecture have helped to carve out important hypotheses regarding the origin of the nervous system. Early contributions envisaged local sensory-effector circuits as first manifestations of the nervous system, referred to as elementary circuits. In these circuits, information transfer would have been mostly vertical, from sensory to effector cells, and mediated by the first neurons. Different kinds of effector cells have been considered for these circuits—from contractile to motile ciliary or even with immune functions.

Later authors instead emphasized horizontal information transfer and envisaged tissue-wide elementary nerve nets as first manifestations of the nervous system. These nets may have acted as endogenous pacemakers, or they may have integrated sensory input for the coordinated control of entire downstream effector tissues—which may have been contractile or bearing motile cilia. These contributions thus led to two major hypotheses for nervous system origins, which survive until today. Following the contractile network hypothesis, the first nerve net originated by division of labour from a network of multipolar mesenchymal cells that were both conductive and contractile (figure 4). Alternatively, the elementary nerve net may have resulted from newly evolving synaptic communication between the basal processes of dispersed sensory-neurosecretory cells that formed part of an epithelium with motile cilia (figure 7).

Recent progress in sequencing the transcriptomes of single cells from entire bodies allows the testing of these hypotheses via comparison of cell type-specific transcriptional profiles within and across species. While this new field of comparative cell biology is just emerging and is still far from a comprehensive understanding of cell type genealogies across the animal kingdom, first observations indicate support for both the contractile network hypothesis and the neurosecretory network hypothesis. For example, while in cnidarians ectodermal neurons and myocytes appear closely related, vertebrates show a close molecular relationship between ventral neural tube neurons and pancreatic secretory cells. Future cross-phyla comparisons of cell types will help in deciding whether these observations can be generalized. As it stands, the data support at least two different origins of nerve nets in different body parts of ancestral metazoans. Also, molecular comparisons will substantiate whether any of these nerve nets are related to the diverse nerve nets found in the enigmatic ctenophores.

According to all prevailing hypotheses the incipient nerve nets enabled some complex feeding or locomotor behaviour, and can thus be seen as an adaptation towards enabling such whole-body movements under the constraint of increasing body sizes. Besides the coordination of movement, such nerve nets would have facilitated information integration in various ways and thus enhanced cognition. First, different external sensory modalities would have fed into the nerve net and triggered one integrated nerve net response to environmental stimuli. Second, via reafferent sensing the nerve net would have also perceived sensory stimuli generated by the animal's own movement and thus integrated internal and external information [83]. This is especially important for large animals that cannot properly move without such integration. Third, the immediate effect of an elaborate nerve net with its increased speed of signalling and multiple synaptic contacts would be to greatly increase the range of habituation and sensitization of different spontaneous exploratory patterns of activity. Overall, the coordinating and integrating effects of the evolving nerve nets cannot be decoupled and sum up to a substantial increase in cognition that accompanied the rise of the elementary nervous system. In conclusion, the origin of the nervous system allowed early animals to ensure behavioural coordination and cognitive capacities in larger multicellular bodies. Without a nerve net, ever-increasing cell numbers would have inevitably led to reduced information flow between cells, and thus to a decrease in cognitive power and integration. The nervous system can thus be seen as an evolutionary response to multicellularity and increasing body sizes in early animals.

## Data accessibility

This article has no additional data.
